# The Chemistry–Process–Structure Relationships of a Functionally Graded Ti-6Al-4V/Ti-1B Alloy Processed with Laser-Engineered Net Shaping Creates Borlite

**DOI:** 10.3390/ma17143491

**Published:** 2024-07-14

**Authors:** D. Seely, M. A. Bagheri, D. Dickel, H. E. Cho, H. Rhee, M. F. Horstemeyer

**Affiliations:** 1 Haynes International, Kokomo, IN 46904, USA; dseely@haynesintl.com; 2 Department of Mechanical Engineering, Mississippi State University, Starkville, MS 39762, USA; bagheri.274@gmail.com (M.A.B.); doyl@me.msstate.edu (D.D.); hrhee@me.msstate.edu (H.R.); 3 Vacuum Process Engineering Inc., Sacramento, CA 95815, USA; 4 School of Engineering, Liberty University, Lynchburg, VA 24551, USA

**Keywords:** additive manufacturing, laser-engineered net shaping (LENS), titanium alloy, boron, microstructure

## Abstract

We quantify the chemistry–process–structure–property relationships of a Ti-6Al-4V alloy in which titanium-boron alloy (Ti-B) was added in a functionally graded assembly through a laser-engineered net shaping (LENS) process. The material gradient was made by pre-alloyed powder additions to form an in situ melt of the prescribed alloy concentration. The complex heterogeneous structures arising from the LENS thermal history are completely discussed for the first time, and we introduce a new term called “Borlite”, a eutectic structure containing orthorhombic titanium monoboride (TiB) and titanium. The β-titanium grain size decreased nonlinearly until reaching the minimum when the boron weight fraction reached 0.25%. Similarly, the transformed α-titanium grain size decreased nonlinearly until reaching the minimum level, but the grain size was approximately 2 μm when the boron weight fraction reached 0.6%. Alternatively, the α-titanium grain size increased nonlinearly from 1 to 5 μm as a function of the aluminum concentration increasing from 0% to 6% aluminum by weight and vanadium increasing from 0% to 4% by weight. Finally, the cause–effect relationships related to the creation of unwanted porosity were quantified, which helps in further developing additively manufactured metal alloys.

## 1. Introduction

Additive Manufacturing (AM) is currently very popular. Design engineers want the advantage of quickly moving information from a 3D CAD file to a finished part using a rapid prototyping paradigm, which AM provides over the traditional bulk material subtractive machining processes or long lead time casting processes. The AM objective is to reduce the iteration time of the design cycle. For metal-based AM, the laser metal sintering process was the first to achieve net shape geometries from 3D CAD files [1], but high porosity levels limited mechanical product applications constrained by fatigue performance [2]. Hence, understanding the role of heterogeneous microstructures and, in particular, porosity becomes an important focus for AM processing. Improvements in process control have brought metal AM parts from the role of prototyping in the design cycle to fully functional and, in some applications, finished parts [3].

The typical metals used for AM include Ti-6Al-4V ([4,5]), 316 SS ([6], Inconel [3], SS410 steel [7], and H13 tool steel [6]. Bhatia and Sehgal (2023) [8] and Gardner (2023) [9] give recent reviews of the AM process–structure–property relationships of different metal alloys. Candidate metals used for metal AM are typically drawn from known alloy systems because much is already known about them from years of use, and quality feedstock is available from which to produce powders. When selecting a material for AM product design, constraints for material properties such as corrosion resistance or high-temperature performance can impose pressure for selection of known alloy families; however, many alloys derive their desirable properties from a specific processing history chosen to achieve a finished microstructure. The advantage of using the AM process is to achieve a desired net shape in a single step. This advantage may be lost if subsequent processing is required to meet a certain property or performance objective.

### 1.1. Adding Multiple Powder Chemistries Is Difficult

Powder-bed methods have since improved process control to minimize pore defects, enabling near-fully dense parts with material properties approximating rapid solidification. Process control development has now been shifted to predictively control solidification rates to achieve a preferential microstructure. With a catalog of process parameters such as laser power, scan speed and pattern, hatch spacing, and beam shape correlated with a predictable microstructure, laser-based additive manufacturing methods present the possibility to selectively tailor location-specific microstructures. Powder-bed fusion methods have typically been limited to a single powder chemical composition.

Blown powder AM methods [10] add a degree of freedom: chemical composition. By integrating multiple feeders, powder metals of varying composition can be blended in the melt pool to achieve an in situ composition of choice. Elemental powders and pre-alloyed master alloys have been used.

The challenge with fusion-based powder metal AM methods is adding a predictable and controlled deposition layer of material. The thickness of the deposition layer is important for tool path planning. Because most AM methods use the planar slice method to build a 3D geometry, variations in slice thickness can be affected. Powder-bed methods maintain control of layer thickness by establishing a new layer of unconsolidated powder whose upper surface is planar with a regularly prescribed offset distance and whose lower surface follows the surface contour of the previous layer’s melt surface. The depth of powder as measured from the upper plane may vary, following process features from the previous layer, such as dips and peaks from hatch pattern variations. Each layer fusion cycle may produce variations in deposition height locally, but deposition layer thickness variations are compensated on successive layer passes.

### 1.2. Laser-Engineered Net Shaping (LENS) Processing Method

Fusion-based blown powder methods are vulnerable to cumulative variations in the deposition thickness, particularly when the chemical composition is changing. Consequently, great care must be taken to ensure that the effect of processing variables on the deposition thickness is characterized. The laser-engineered net shaping (LENS) method, first developed by Atwood et al. (1998) [11], makes use of a strategy to minimize cumulative departures from a planned geometry called a deposition zone. Powder flow is directed into the deposition zone such that the mass flow varies with respect to the relative location along the laser beam axis. If too much material is added to the melt pool on a given deposition pass (a condition called overbuilding), then successive layer surfaces will intersect the deposition zone higher along the beam axis and receive less powder mass flow, therefore reducing the material addition rate. If too little material is added to the melt pool (a condition called underbuilding), then successive layer surfaces will intersect the deposition zone lower along the beam axis and receive more powder mass flow, therefore increasing the material addition rate. This form of intrinsic process feedback control provides flexibility in using an open loop prescribed motion tool path. Deposition parameters are chosen to maintain a mass addition rate with an expected deposition thickness that maintains the build within the center of the deposition zone. Examples of using the LENS method with titanium alloys include Raji and Popoola (2024) [12] and Antolak-Dudka et al. (2024) [13], who recently analyzed the process–structure–property relationships in chemomechanical environments, Eliaz et al. (2020) [14] who repaired defected structural components, and Izadi et al. (2020) [15] who conducted a parametric study on the process parameters.

### 1.3. Functionally Graded Material via the LENS Method

In this paper, the LENS method is employed to create a functionally graded material (FGM) to provide an understanding of the fabrication complexities related to the chemistry–process–structure relationship. LENS was first developed by Sandia National Laboratory in 1998 [11] and uses a powder-feed laser-energy deposition technique [16]. The powder is rapidly melted by the high-energy laser and quickly cooled, creating a solidified layer. Since LENS deposits a layer in a point-to-point manner through the nozzle injection, it can yield material with gradually changing compositions and properties [16,17]. The use of two different materials in a mechanically coherent system can produce a condition of incompatibility at the interface between the two materials, thus affecting the deposition rate and, in turn, the control system, causing uncertainties in the resulting microstructures. Incompatibility can arise during fabrication and manifest different microstructures that reduce the properties of interest and, hence, the performance [18]. When the selection of each material is constrained by a functional material requirement in a specified region, then the designer must find a means to maintain compatibility between the regions. One approach is to arrange the material in such a way that the aggregate material properties change slowly from one region to the other. This is called an FGM [17,19].

Our specific intent was to add Ti-1B into a Ti-6Al-4V alloy in an FGM manner. Borides of titanium have high modulus (4× greater), high hardness (3× greater), and coefficients of thermal expansion compatible with titanium alloys. The high hardness and modulus of titanium monoboride suggest that it would be a good candidate for the hard layer of a composite armor system. Because of the compatible thermal properties, delamination of adjacent layers of titanium and TiB by a large temperature range of fabrication processes may be minimized [20,21,22].

There have been different methods by which TiB has been added to a base alloy. Lepakova et al. (2000) [23] prepared titanium boride phases by a self-propagating high-temperature synthesis. Wang and Thompson (1999) [24] produced a TiB_2_ plate by self-propagating high-temperature synthesis and dynamic compaction (SHS-DC) with high density (99.3%) and large grain size (18 ± 3 μm). TiB has been fabricated by elemental powder spark plasma sintering (SPS) [25,26], laser coating [27], LENS pre-alloyed Ti-6Al-4V, and elemental boron [28]. More recently, Attar et al. (2014) [29] used selective laser melting processing to fabricate Ti-TiB composite. Bermingham et al. (2015, 2018) [30,31] applied wire arc additive manufacturing by adding elemental boron and trace lanthanum hexaboride (LaB_6_) to Ti-6Al-4V. In particular, with the addition of LaB_6_, TiB particles were formed by the decomposition of LaB_6_ mixed with titanium to TiB and La_2_O_3_. Yang et al. (2021) [32] also used wire arc additive manufacturing to fabricate Ti-6Al-4V with cyclic gradient distribution of TiB whiskers to improve the plasticity of the alloy, mitigating its high strength. A powder-bed fusion with electron beam melting processing was used for Ti-6242S-1B to apply a rapid cooling rate to fabricate dispersed fine TiB particles more uniformly [21,33]. Other studies also used the laser powder-bed fusion method for titanium alloy-TiB nanocomposites [34,35]. Dong et al. (2020) [36] applied a selective laser melting additive manufacturing method to process Ti-TiB composite. Liu et al. (2023) [37] used laser-directed energy deposition for TiB-reinforced Ti-6Al-4V composite. Finally, Zhang et al. (2018) [38] provided a review of electron beam AM methods of titanium alloys.

**Figure 1:**
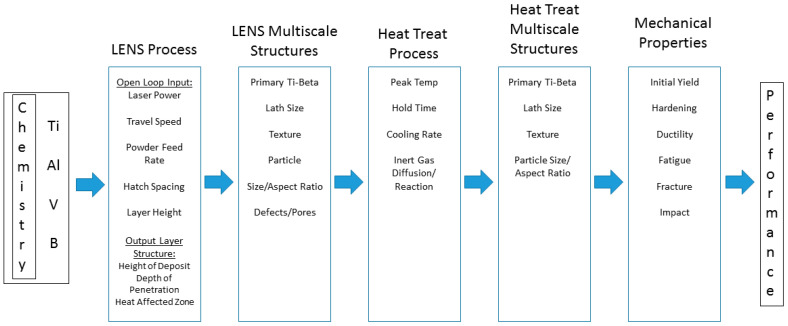
Chemistry, process, structure, property, and performance diagram for the laser-engineered net shaping (LENS) additive manufacturing process.

The contribution herein is the complete thermomechanical description of the complicated multilayered LENS chemistry–process–structure relationships of a Ti-6Al-4V and TiB functionally graded material of which arose “Borlite”, a eutectic structure containing orthorhombic titanium monoboride (TiB) and titanium. The titanium-based FGM was made to quantify the multiscale process–structure relationships related to liquid/solid transformations, solid-state phase transformations, and chemical equilibrium states for a graded material (see Figure 1) for the chemistry–process–structure–property relationships. The next section describes the materials processing of the LENS method with the characterization of the multiscale structures. Section 3 describes the microstructural results, and Section 4 offers a description of the important process–structure features. Finally, some conclusions are drawn in Section 5.

## 2. Processing Method of LENS for the Titanium-Aluminum-Vanadium-Boron Functionally Graded Materials

**Figure 2:**
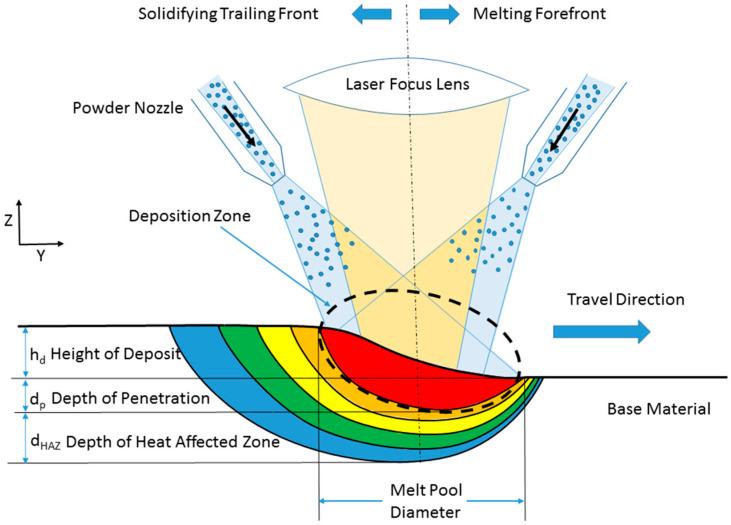
Schematic of the traveling deposition zone of the LENS blown powder method. The base material is either a starting material or a previous layer and is fixed. The deposition head travels to the right relative to the base material, as shown in this schematic. The dotted line represents the melt region, where the red color is the hottest region under the laser focal spot. The amount of the powder filler material determines the height of the deposition pass. The melt pool diameter is a function of laser power and travel speed, which in turn affects the depth of penetration and the heat-affected zone. The melting forefront experiences a different thermal history than the solidifying trailing front that generates a different microstructure.

Two pre-alloyed spherical powders were used to make 25.4 mm × 25.4 mm specimens with compositions of Ti-6Al-4V and Ti-1B (1 wt.% boron) on a 1/4-in thick commercially pure titanium base plate with a LENS 750 machine. The hatch spacing was 0.381 mm, and the travel speed was 8.5 mm/s. The powder flow was coordinated by the mass flows proportioned between the two powder feeders, as shown in Figure 2. Note from Figure 2 that the deposition height, h_d_, is different from the depth of penetration, d_p_, and depth of the heat-affected zone, d_HAZ_. A functional grade in composition was prescribed in eight layers. Layer 1 started with 100% Ti-6Al-4V powder from Feeder #1. Layer 2 decreased the flow rate of Feeder #1 to 90% Ti-6Al-4V and raised the flow rate to 10% Ti-1B powder from Feeder #2, and so on through Layer 8. As such, the amount of Al and V decreased with increased layer number, while the amount of B increased. By trial and error, the operator selected a laser power level that provided satisfactory melting for both individual powder types. Then, the same laser power and travel speed settings were applied to all eight composition layers.

**Figure 3:**
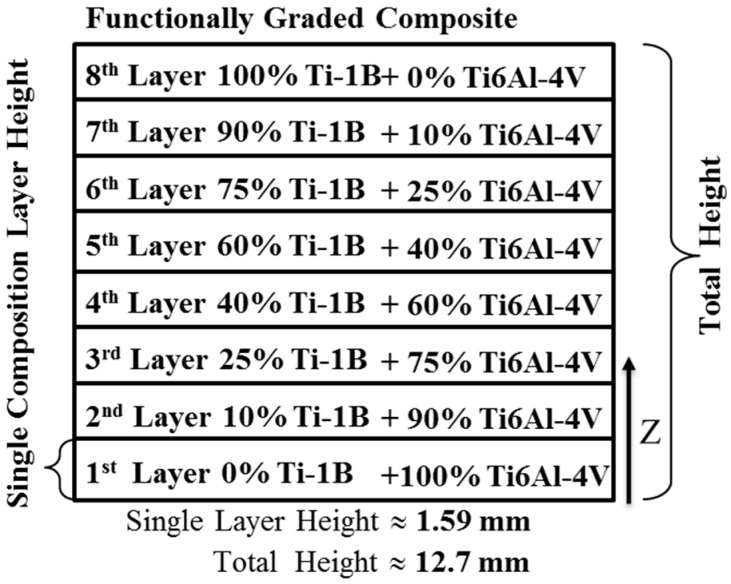
Schematic showing the layer composition of the Ti-6Al-4V/Ti-1B FGM produced by LENS.

The multiple-layer FGM described herein is illustrated schematically in Figure 3, illustrating the different amounts of TiB that were included in each layer, each approximately 0.159 mm thick. Six passes with a hatch pattern created each of the eight layers of differing composition rotated 45 degrees relative to the previous pass. Each pass deposited between 200 and 400 μm of material.

**Figure 4:**
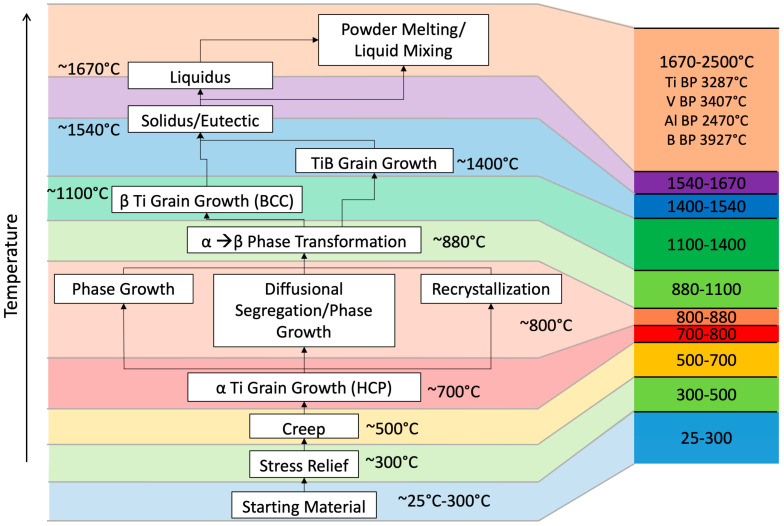
Schematic of different “events” that occur in the titanium functionally graded material as the temperature increases in the direction of the melting forefront. Please note that multiple multiscale structures can arise during the complex temperature history.

**Figure 5:**
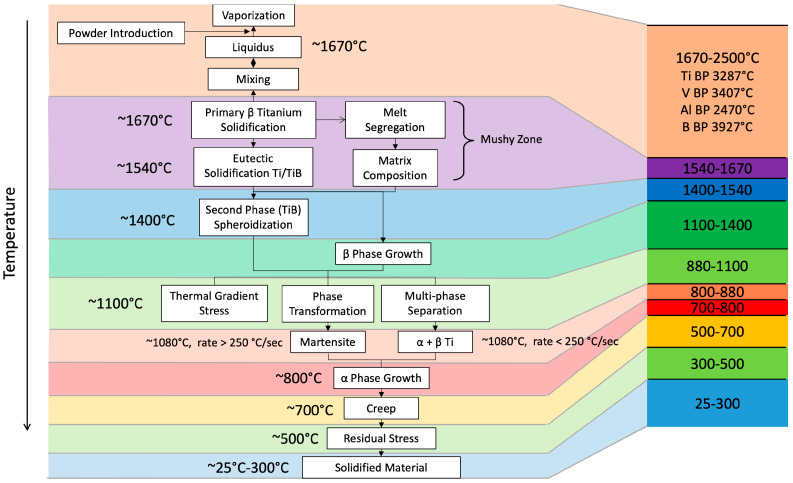
Schematic of different “events” that occur in the titanium functionally graded material as the temperature decreases in the direction of the solidifying trailing front. Please note that multiple multiscale structures can arise during the complex temperature history.

Changes in the bulk chemical composition of the deposited material affect the deposition process features at the macroscale. Figure 4 and Figure 5 show schematics of the chemical compositional and structural changes as temperatures increase and decrease respectively. Figure 4 shows changes that take place in the melting forefront as the temperature increases and Figure 5 shows changes in the solidifying trailing front as the deposition zone temperature decreases. Hence, one material point may experience multiple temperature excursions and thus exhibit different structural changes such as phase changes, recrystallization, grain growth, segregation, creep, and finally, stress relief (c.f., [39]). Different macroscale and microscale structures arise that are associated with the different temperature levels that a material point will experience during the LENS process.

## 3. Results

### 3.1. Process–Structure Relationships Related to Porosity

The first goal of process control for blown powder methods such as the LENS process is to minimize the process-related porosity; in other words, to quantify the macroscale structures that determine the mechanical properties. Porosity, in the LENS process, has two major forms: gas bubbles present in the melt pools during solidification, typically spherical, and deposition-related lack of fusion defects that are typically irregular in shape but flattened in the direction of the deposition plane. Gas bubble defects are introduced to the melt from pre-existing pores trapped in the feed powders, process-related vaporization, or gas entrained by blown powder injection into the melt pool. Because Argon is used in the LENS process, once formed, gas bubbles are maintained in the melt pool during convection and even through solidification. Deposition-related lack of fusion defects can arise from poor spacing between adjacent hatch fill lines, insufficient laser power available to melt both incoming powder and substrate, powder overfill where too much powder is added for the selected laser power, and the mismatch between the laser spot size and melt pool diameter.

**Figure 6:**
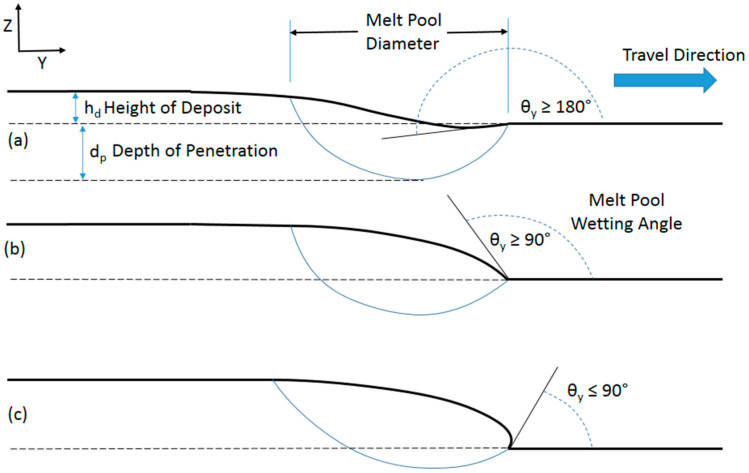
Three different angles of the leading edge of the LENS deposition zone: (**a**) underfilling inducing the greatest depth of penetration, (**b**) acceptable filling, and (**c**) overfilling inducing the least depth of penetration and admitting the lack of fusion defects.

**Figure 7:**
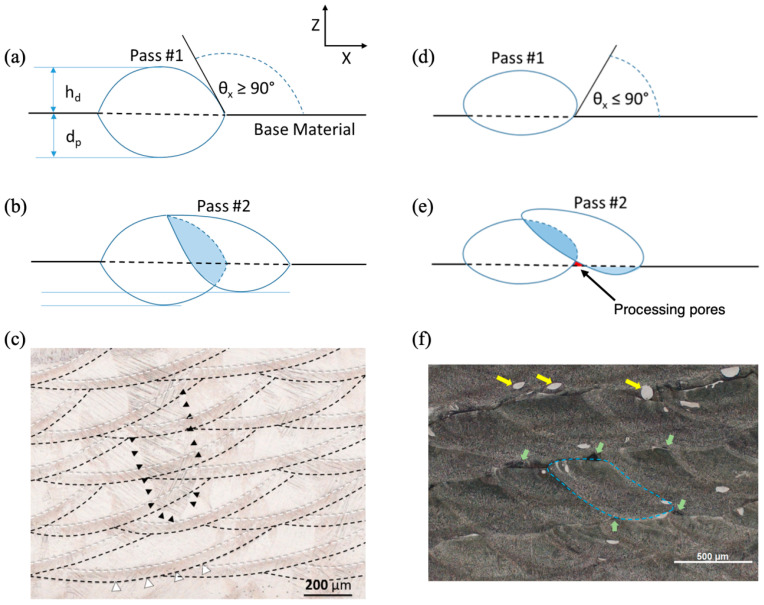
Schematic of the single deposition layer sequence of passes (**a**,**b**) an acceptable LENS process and (**d**,**e**) an unacceptable LENS process. (**a**) First deposition pass with acceptable penetration and fill where melt pool contact angle (θ_x_ > 90°). (**b**) A second pass shows an acceptable hatch-remelted region with an appropriate depth of penetration. (**c**) Bright-field optical micrograph showing successful deposition. Black triangles indicate a single prior titanium β-grain boundary passing through several deposition layers. Black dashed lines indicate start of phase transformation. White dashed lines indicate complete phase transformation in heat affected zone. Regions between experience partial phase transformation. White triangles show a region of overlapping heat affected zones where incomplete phase transformation occurs twice. (**d**) The first pass with unacceptable penetration and fill with melt pool contact angle (θ_x_ < 90°). Please note that (**d**) shows a lack of penetration and overfill, which produces a leading-edge overhang. (**e**) A second pass that allows a pore to nucleate due to lack of penetration, (**f**) A Bright-field optical micrograph showing 4 layers of actual LENS deposition displaying pore distribution caused by lack of penetration. The yellow arrows point to unmelted Ti-6Al-4V powder, the green arrows point to processing pores, and the blue dashed line represents a single deposition pass.

Figure 6 illustrates the three different angles that can arise from the leading edge of the deposition zone. Critical to porosity here is when the angle is less than 90°, as shown in Figure 6c. This angle of less than 90° can arise when overfilling of the powder occurs with respect to the travel speed of the pass as it is laid down. As Figure 6 illustrates, what one really desires is to have an underfilling or acceptable filling powder rate combined with the appropriate travel speed to garner a greater depth of penetration. Figure 7 compares cases when no pores will arise from acceptable filling or underfilling and when pores will arise from overfilling. Note the lack of depth of penetration in the cases of overfilling. Figure 7c,f also show bright-field optical micrographs of the material, showing the difference between the two cases when pores are present and when pores are not present.

**Figure 8:**
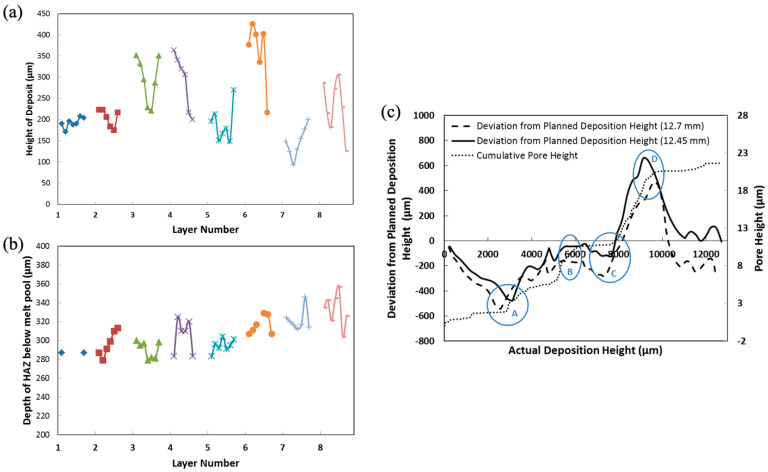
For each deposition layer pass in the eight-layer FGM, (**a**) the height of deposition pass, as measured by HAZ to HAZ and melt pool bottom to melt pool bottom between successive deposition passes. Symbols show successive passes within the same layer colors and symbols are from the same layer and correlate between (**a**,**b**). (**b**) The depth of the heat-affected zone (HAZ) as measured from the bottom of the melt pool. (**c**) Error accumulation between the planned and actual deposition height. Points A, B, C, and D indicate the control loop in situ modifications because of the porosity level.

Since porosity will arise mainly when the depth of penetration is too shallow, it is worth determining the cause–effect relationships to create a certain depth of penetration. Bright-field optical micrographs reveal the perimeter of the heat-affected zone (indicated by white and black dashed lines in Figure 7c) and the boundary of the melt pool (indicated by the blue dashed line in Figure 7f). From the spacing between either the melt pool bottom or the heat-affected zone (HAZ) bottom of successive layers, the height of each deposited layer was determined. Figure 8 shows the actual deposition process features for each deposition pass in the eight-layer FGM. As a reminder, each layer, having a different composition, was formed from six separate passes, each depositing material with a consistent composition for that layer. Figure 8a shows the deposit height derived from measuring the distance either between successive HAZ patterns or the melt pool boundary. Figure 8b shows the depth of the HAZ as measured from the melt pool boundary. Recall that in the LENS blown powder AM method, each deposited surface was not truly level and that each deposition pass had a different thickness depending on the in situ process conditions, as shown in Figure 8a. Consequently, the errors in deposition height accumulated during the build, as shown in Figure 8c. Error here refers to the difference between the actual total build height and the planned build height. The distribution signifies that the control loop is trying to adjust the previously laid powders. Note in Figure 8a that the deposition height ranged from 100 microns to almost 450 microns. These variations can lead to overfilling and thus lead to porosity. A study of process defects collected using X-ray computed tomography (CT) correlated defect location, size, and shape to the deposition process features is shown in Figure 8. Because the hatch spacing, powder mass flow rate, and laser power remain constant, the height of the deposit will correlate with the depth of penetration. The depth of penetration (d_p_) cannot be measured directly in a multilayer deposit since the original reference surface is remelted, so an ambiguity arises for d_p_. Therefore, the height of the deposit (h_d_) was chosen to capture the trend for the depth of penetration. Figure 8c shows two different plots based on assumptions of the planned deposition height, which was not originally given to the processing design. The measured final height was 12.45 mm, but if that was planned, the deviation is shown in Figure 8c. Alternatively, the original planned height was 12.7 mm, so the deviation was also plotted with that assumption in Figure 8c. As such, we do not really know what the planned height was nor how the control loop made decisions in the LENS processing, but clearly, when we compare both data to when the cumulative pore height increased, there were four specific alterations during the build process. Points A, B, C, and D indicate the control loop in situ modifications because of the porosity level. Hence, the deposition height affected the porosity, and the porosity affected the laying down of the next layer particle volume.

**Figure 9:**
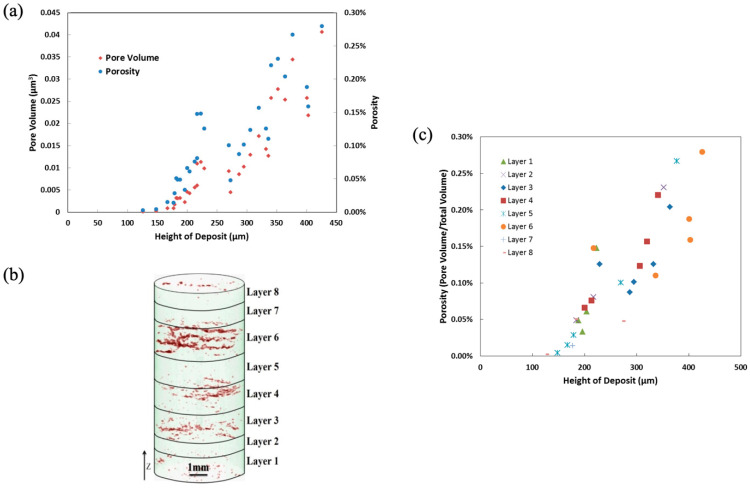
Process defects detected through X-ray computed tomography (CT). At fixed hatch spacing, the effect of the height of the deposit (h_d_) is shown versus (**a**) total process pore volume per pass for each layer. (**b**) An X-ray CT volume rendering showing process pore distribution (colored red) through the volume of a cylinder spanning all 8 layers. (**c**) Porosity per pass for each layer.

**Figure 10:**
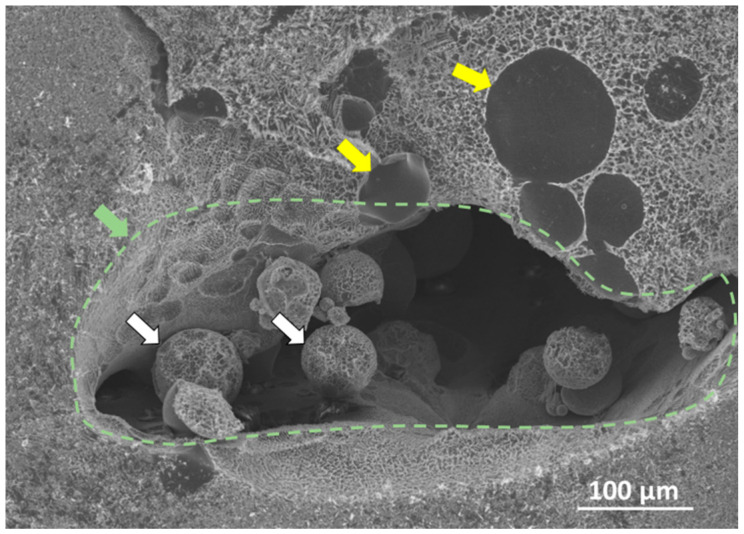
A processing defect is shown in a scanning electron microscope (SEM) image of a region in Layer 6. The yellow arrows point to unmelted Ti-6Al-4V powder; the white arrows point to unmelted Ti-1B powders; the green arrow and the green dashed line outline a single process pore corresponding to green arrows in Figure 7f.

Figure 9 shows the variation in porosity throughout the sample. Figure 9b shows the distribution of pores throughout the sample, determined by CT. The number density and volume of pores both increased with a lot of scatter with increased deposition height (Figure 9a), but the total process pore volume shows a clear increase as the height of deposition increased, as shown in Figure 9c. All the data reduction shown in Figure 9 was garnered from correlating the CT data with optical microscopy-derived deposition features from Figure 8. Figure 10 shows a local pore from a scanning electron microscope (SEM) image illustrating the distribution of porosity near unmelted regions, which then, in turn, incurs a low depth of penetration. Note that when comparing the pore in Figure 10, the image matches the geometrical structures shown in the schematics of Figure 7. Hence, the pore shown in Figure 10 arose from the second pore type mentioned earlier, which is deposition-related and irregular from the lack of fusion.

### 3.2. Process–Structure Relationships Related to Titanium Microstructures

**Figure 11:**
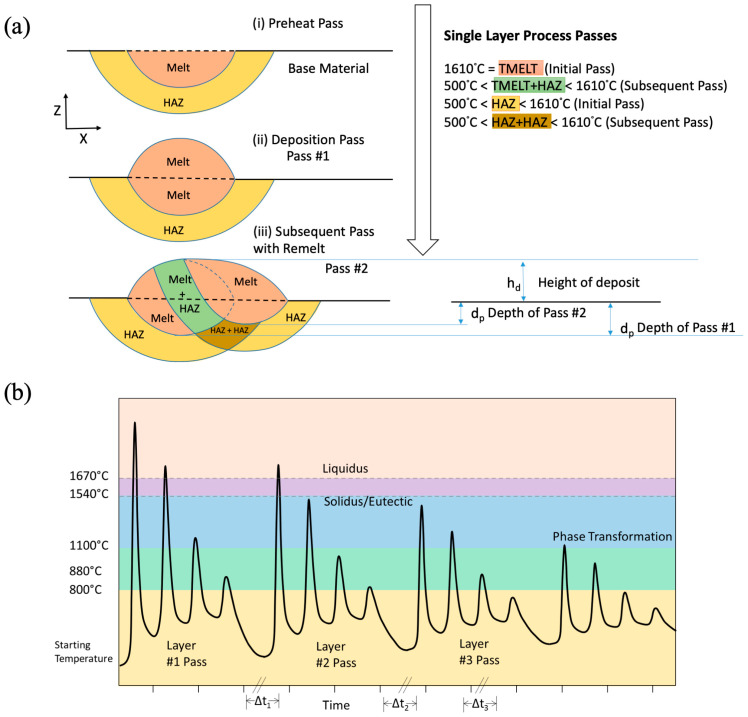
For acceptable passes, the schematic (**a**) shows a preheating pass (i), a single deposition pass (ii), and a subsequent pass (iii) illustrating the melt zone, heat-affected zone (HAZ), and base material. Please note that the depth of a particular pass can vary. We also show here that in just two lines of one pass, we have a potential for four unique temperature history-dependent microstructures, all with the same original chemistry. (**b**) A material point undergoes multiple excursions through the melting and phase transformation zones for titanium alloys due to 4 different passes with multiple lines.

Because different material points will experience different thermal histories throughout the LENS building process, it is important to understand the convolution of complex thermal history with the complicated microstructural morphologies. For example, Figure 11a shows simply one preheat pass, denoted as (i), in which the base material will incur a melt and HAZ. Once the first deposit is laid down (ii), a powder will melt on top of the base material. When the next line of the pass (iii) is laid down and melted, it affects the previously melted powder in a complex manner. Note in Figure 11 that four different microstructures (melt, HAZ, Melt + HAZ, and HAZ +HAZ) will exist because of the four different thermal histories experienced in the case of just two passes.

**Figure 12:**
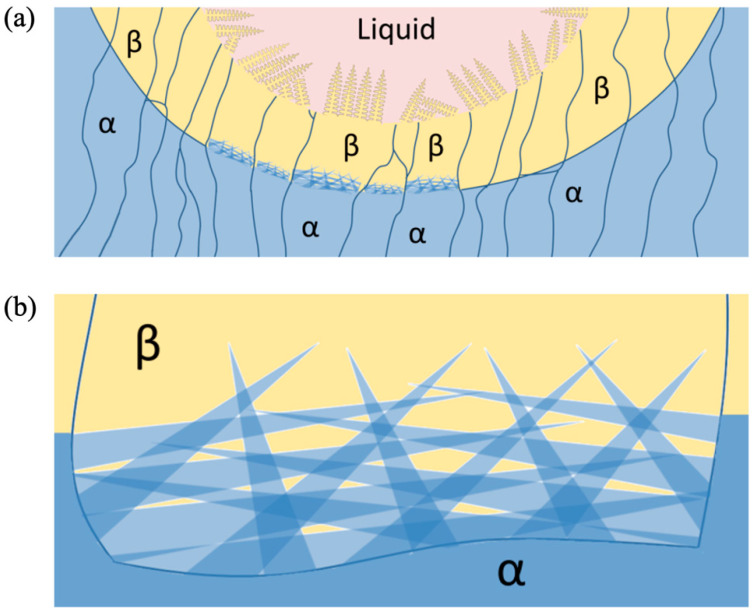
(**a**) A schematic of the events: liquid to solid β, solid β to solid α. (**b**) A schematic of acicular morphology of α transformed at high cooling rates.

The processing also causes complex microscale features due to the thermal cycling acting as different heat treatments once the original material was laid down. Figure 11b shows a schematic of a possible temperature excursion experienced by a material point as it is melted, possibly several times, and part of several different HAZ with differing intensities. Overall, this hypothetical material point would experience 16 thermal cycles at different temperatures, assuming the HAZ is approximately four passes thick. Assuming a Ti-6Al-4V chemistry, this point would experience melting and solidification three times, move through the mushy zone once, pass eight times from above β-transus temperature, pass 12 times through the phase transformation zone, and return five times to the α + β stability zone. At this point, the final microstructure would retain history-dependent features and an associated morphology. The solidification front structure in Figure 12a depends on the cooling rate, which gives rise to a particular dendrite cell size spacing, grain size, and eutectic morphology. After solidification, the liquid to solid β-phase titanium then turns to solid phase α-titanium. Figure 12b shows the acicular morphology of the α-phase titanium that transforms from the β-phase titanium. The heating and cooling rates are so high for laser-induced heating that this acicular morphology arises for the α-phase titanium [40]. The complexity of the microstructure only increases when all the passes and layers are added.

### 3.3. Chemistry–Process–Structure Relationship Related to Boron Additions

**Figure 13:**
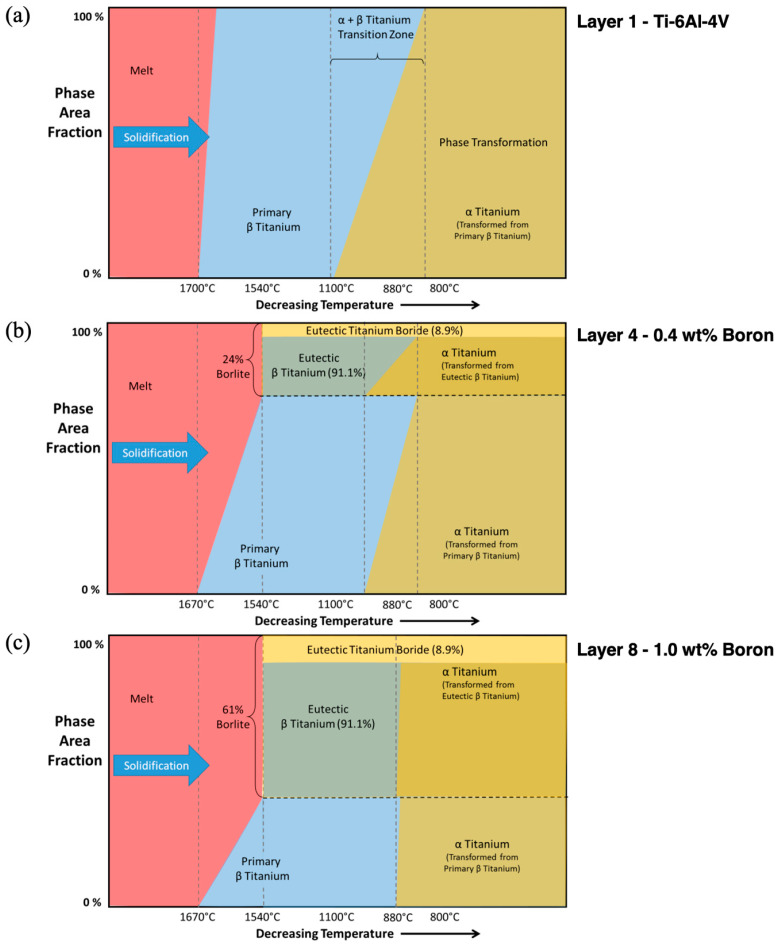
Phase area fraction expressed as a function of decreasing temperature through solidification and phase transformation for (**a**) Layer 1Ti-6Al-4V, (**b**) Layer 4—0.4 wt.% Boron, and (**c**) Layer 8—1.0 wt.% Boron.

The microstructure will be different throughout the different layers because of the varying chemistry. Figure 13 illustrates how the phase area fraction changes during solidification and solid-state phase transformation for varying concentrations of Ti-6Al-4V and Ti-1B. Recall that as the layering increases from the first to the eighth layer, the boron amount increases from 0% to 1%, and the aluminum and vanadium amounts decrease from 6% and 4%, respectively, to 0%. For Layer 1, shown in Figure 13a, where no boron is present, the solidification temperature range is narrow, approximately at 1700 °C, and the α + β-phase transformation range spans from approximately 1100 °C to 850 °C. For Layer 4 at 0.4% boron composition, the solidification range covers a wider temperature range from approximately 1700 °C to 1540 °C because of the eutectic reaction of boron with titanium. We introduce the term “Borlite” to define this material.

Borlite is a eutectic structure containing ~8.9% orthorhombic titanium monoboride (TiB) by volume and ~91.1% Titanium. The final area fraction of Borlite is 24%, of which 8.9% is eutectic TiB and 91.1% is eutectic titanium. Note that the reduction in aluminum and vanadium narrows the α + β-transition zone for both the primary and eutectic titanium. For Layer 8 with 1.0% boron composition, the solidification temperature range remains wide, but the liquidus temperature reduces to 1670 °C while the solidus remains at 1540 °C. The final area fraction of Borlite is 61%, and the dual solid-state phase transition zone has narrowed to 882 °C. In summary, Figure 13 illustrates the expected phase area fractions correlated with the composition, chemistry, and morphology.

**Figure 14:**
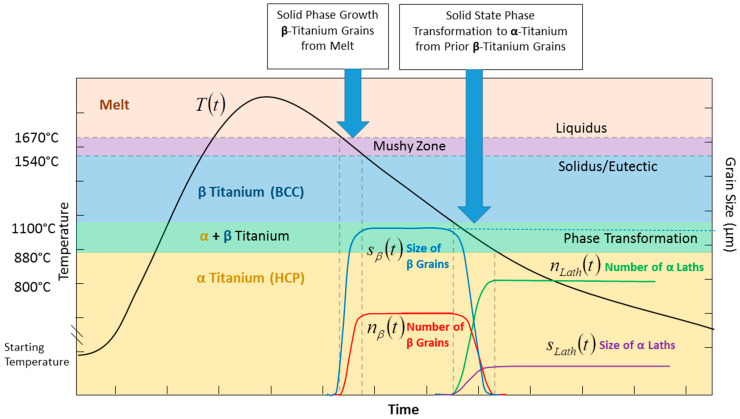
Temperature-time history for single pass showing grain size and number and alpha lath size and number.

The stereology of the microstructural features generated in a single temperature passage through the phase transition zones (shown in Figure 13) is dependent on the cooling rate. In Figure 14, the black line shows the temperature profile of a point increasing into the melt zone and then decreasing through solidification and phase transformation. Figure 14 illustrates how the number density (η_β_) and size (s_β_) of the β-titanium grains increase; later, the η_β_ and s_β_ decrease as the number density (η*_Lath_*) and size (s*_Lath_*) of α-laths increase consuming the β-grains. For a given volume fraction of β-titanium grains, the number and size will be determined by the cooling rate.

**Figure 15:**
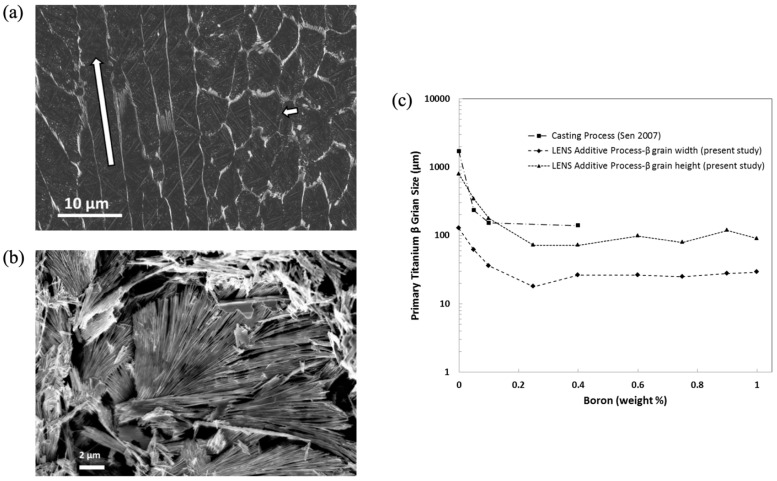
Scanning Electron Micrograph (SEM) of titanium boride eutectic regions < 0.1% boron. (**a**) Long white arrow indicating adjacent primary titanium dendrite arms decorated by eutectic TiB. Short white arrow shows the transverse section of adjacent dendrite arms enlarged in (**b**) after deep etching to remove the titanium matrix. TiB whiskers are shown following primary dendrite arm cells. (**c**) Primary titanium β-grain size as a function of boron composition comparing additive manufacturing (LENS) results from the present study to that of casting (skull melt process) [41]. Please note that the initial grain size is related to the grain size of the base material, which increased due to heating.

Sen et al. (2007) [41] studied the influence of small additions of boron on the primary solidification of Ti-6Al-4V by casting via the skull-melting process. They showed a grain refining effect for boron additions up to 0.1 wt.% composition. Figure 15c shows the β-titanium (BCC) grain size with respect to boron concentration, comparing the casting process of Sen et al. (2007) [41] to our work with the LENS. Please note that the trends are very similar, although the heating and cooling rates are different from those of a skull-melting casting versus those of a laser-based approach like LENS.

## 4. Discussion

### 4.1. Initial Titanium Beta Grain Size

The multiscale structure of importance is the β-titanium grain size. Figure 15 shows SEM pictures of the TiB in the eutectic region of Layer 2 when the boron was less than 0.1%. Solidification of the melt pool in the LENS process is predominantly directional, dendritic, and epitaxial, inheriting the grain orientation of the seed grains at the solid–liquid interface, as shown in Figure 15a. The primary features to emerge during solidification are the β-titanium (BCC) grains. These are the first to solidify from the melt. The number of β-grains that result when the melt pool solidifies is correlated with the number of seed grains in the solid decorating the solid–liquid interface. Figure 15b shows the acicular needles that arise because of the rapid cooling rate. For the first layer deposited on a mill-annealed commercially pure base plate (average grain size ~46 μm) and a melt pool diameter of 780 μm, there are approximately 40 grains spanning the solid–liquid interface of a two-dimensional slice of the melt pool. However, the β-grain size increased in the solid material near the melt pool interface due to heating-induced grain growth, therefore increasing the seed grain size as shown in Figure 15c. The dendritic growth is solidification rate dependent. The spacing of the primary dendrite arms is proportional to the solidification rate [42]. Where the seed grain size is larger than the primary dendrite arm spacing, multiple dendrites nucleate from the same parent grain surface (in the case of a smooth solid/liquid melt interface) or are retained (in the case of remelting that follows previous dendritic structures). In the absence of chemical content to cause melt segregation, dendritic growth of the solid phase consumes the liquid phase until adjacent dendrites merge, leaving little trace of secondary dendrite arms. The solidification direction follows the temperature gradient in the melt pool. While the solidification direction at the bottom of a stationary LENS melt pool is directed upward, the solidification direction of a translating melt pool is horizontal at the melt pool surface sides and trailing edge, such that total solidification orientation is a mixture of vertical and horizontal directions. However, because the melt pool from the deposition of a new layer penetrates the previously deposited surface, a portion of the horizontal dendrite orientation is lost to remelting. The degree to which horizontal solidification features are eliminated depends on the depth of penetration of the new layer melt pool. Consequently, the structure that emerges from the LENS process often resembles columnar grains in casting. In titanium, the primary solidification phase is body-centered cubic, the preferred solidification orientation of which is the [100] direction. Pure titanium is an allotropic metal with a body-centered cubic structure stable at temperatures below melting (~1670 °C) and above 882 °C and a hexagonal close-packed structure stable below the phase transformation temperature of 882 °C. Therefore, although a [100] texture is established in the direction of solidification, the resulting texture is dependent on the transient transformation behavior of the specific titanium alloy employed.

It can be assumed that the melt pool has a homogeneous composition when feed powders are of similar composition. However, making use of a dual powder feeder to achieve in situ mixing may result in compositional gradients in the melt pool.

### 4.2. Borlite Titanium/TiB Eutectic Region

In this study, all layer compositions are hypoeutectic with respect to boron, with the highest concentration being approximately 1 wt.%. Boron forms a eutectic reaction with titanium at a composition of approximately 1.64 wt.% and a temperature of 1540 °C. Figure 13b shows the sequence phase volume change during solidification of Layer 4 with 0.4 wt.% boron. Under near-equilibrium conditions, the BCC titanium solid phase grew until it reached 76% volume. At that point, solidification followed the eutectic reaction, and the remaining 24% liquid formed a solid phase (Borlite). The morphology of the TiB in the Borlite varies from needle-like whiskers at high solidification rates to plate-like sheaves at lower rates. The term Borlite is inspired by the term Pearlite used to describe a eutectoid structure in carbon steels. The phase volume ratio of cementite to ferrite remains constant, but the morphology of the cementite can vary sufficiently to be observed in optical microscopy, causing observers to identify one optically distinct form as Pearlite and another as upper and lower Bainite.

The presence of boron in the melt influences the solidification structure of titanium. At low concentrations (~0.1 wt.%), boron remains in the liquid phase as titanium dendrites grow and thicken. The space between the secondary dendrite arms is filled with primary titanium. By the time that boron has been enriched in solution sufficient to reach the eutectic composition, the primary titanium dendrites have reached a columnar form. When the eutectic solidification initiates, it occurs in the liquid volume space between the primary dendrite arms of the primary titanium phase.

As the starting concentration of boron increases, the eutectic solidification initiates earlier in the primary dendrite solidification sequence. The Borlite region occupies more space between adjacent primary dendrite arms, as illustrated in Figure 15a—Layers 2 and 3 with concentrations of 0.1 and 0.2 wt.% are shown in this structure.

**Figure 16:**
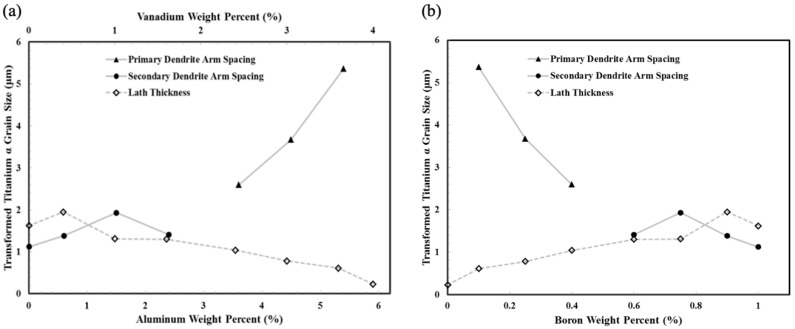
Transformed titanium α-lath thickness, average primary and secondary dendrite arm spacing measured with respect to (**a**) aluminum and vanadium composition by wt.% and (**b**) boron composition wt.%. When both primary and secondary dendrite arm spacing is observed, the smaller of the two are plotted.

At some point, the starting concentration of boron is sufficient to interrupt the primary titanium dendrite growth at the early stage of secondary dendrite arm formation. The melt concentration of boron reaches the eutectic composition before the space between the secondary dendrite arms can be filled with primary titanium. The remaining space is filled with Borlite, extending out to the interdendritic space. This is observed in sparse locations starting in Layer 3 with 0.2 wt.% boron, increases in Layers 4 and 5, and becomes dominant in Layers 6, 7, and 8. The variation is attributed to the difference in cooling rate through the melt pool, which will, in turn, influence the primary and secondary dendrite spaces. Figure 16b shows the average primary and secondary dendrite arm spacing with respect to changes in boron composition.

Grain refinement is observed with the smallest boron addition occurring in Layer 1 at 0.1 wt.%. The size of the β-titanium grain perpendicular to the growth direction decreases. The effect that boron seems to have is to segregate primary dendrite cores at the solid/liquid interface. This segregation seems to permit competition between dendrite cores and prevents the domination of larger grains in the melt pool. Without boron additions, the primary β-titanium grains propagate through successive deposition layers. With 0.1 wt.% boron, primary β-titanium grains can still propagate through successive deposition layers, but the dimension of the β-grain in the deposition plane decreases. Where Layer 1 has 7.5 grains across the melt pool, Layer 2 has 26 grains. They are still primarily oriented normally to the deposition surface, imparting an initial [110] texture. Figure 15c shows the reduction in primary titanium β-grain size with respect to changes in boron concentration for LENS deposited material and casting, as reported by Sen et al. (2007) [41], who used the skull-melting process.

### 4.3. Phase Transformations

**Figure 17:**
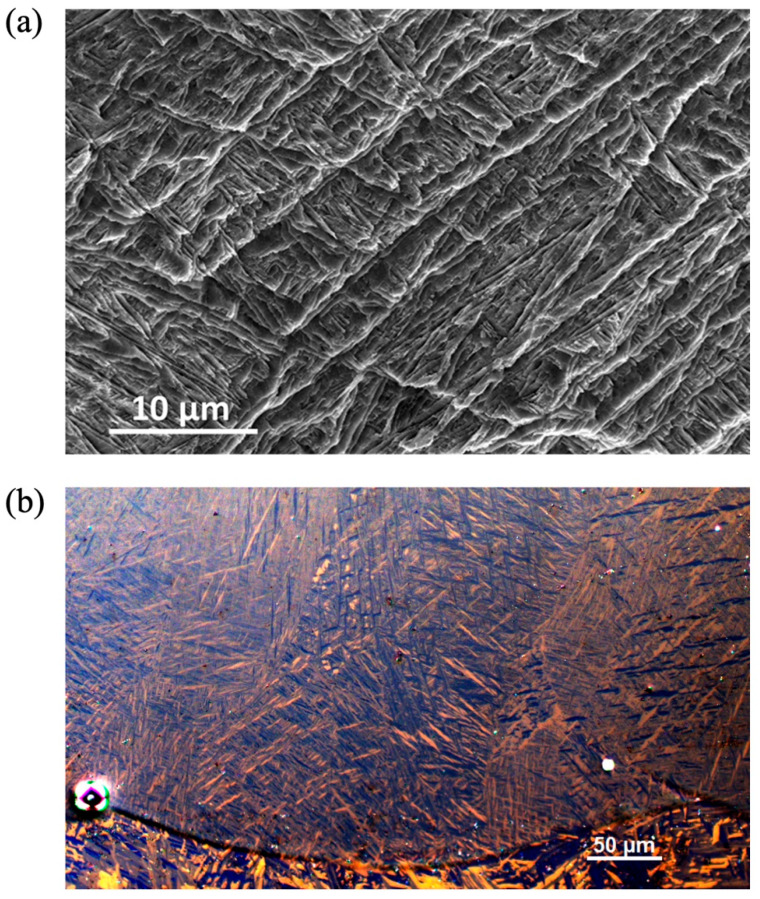
(**a**) A scanning electron microscope image of LENS deposited Ti-6Al-4V region from Layer 1 after deep etching with nitric acid. Transformed titanium α-laths forming a wedge-shaped pattern. (**b**) A polarized light optical micrograph of Ti-6Al-4V Layer of similar region.

The third microstructural feature of relevance is the phase sizes after transformation. Figure 16a shows the change in transformed titanium α-lath thickness with respect to the aluminum and vanadium composition, which decreases as boron concentration increases. The final lath thickness decreased from 2 μm to 0.2 μm as vanadium and aluminum concentrations increased. The alloy chosen to blend with the boron was Ti-6Al-4V with six percent weight aluminum and four percent weight vanadium. Both elements form a solid solution with titanium. As a binary alloy, aluminum additions to titanium stabilize the HCP phase and increase the phase transformation temperature. Vanadium naturally forms a BCC structure; consequently, as a binary alloy with titanium, vanadium stabilizes the BCC phase and reduces the transformation temperature. When used in combination, aluminum, and vanadium produce a heat-treatable titanium alloy capable of producing a host of multiple-phase microstructures. Most heat treatments involve soaking the material at a temperature where alloying elements can segregate from the starting β-titanium grain size into α- and β-titanium phases by diffusion, then controlled cooling refines the remaining β-titanium phase through transformation to α-titanium. When the Ti-6Al-4V alloy is used in AM processes, the final microstructure is determined by the time and temperature history of cooling through the transformation temperature range. The material can be modified by subsequent heat treatment, but the initial β-titanium grain size and texture are locked in by the solidification structure. In Layer 1, the average β-titanium grain diameter was 110 μm. For comparison, the Ti-6Al-4V mill-annealed rolled plate had a grain size of approximately 2–4 μm. Ti-6Al-4V descending through the transformation zone at a high rate (>250 °C/s) undergoes martensitic transformations (so-called because they are considered diffusionless). The crystallographic structure is an α-HCP titanium. The β-titanium grain transforms into a nearly plate-like lath structure of the α-HCP titanium in 12 hexagonal variants {110} _β_//(0002) _α_,<111> _β_//<$11\bar{2}0$> _α_. The thickness of these plates is consistent and can be traced from one prior β-grain boundary edge to the other. Interpreting the distribution and morphology of α-titanium plate-like laths from polished optical micrographs is challenging. For bright-field imaging, etching is required to provide sufficient contrast to distinguish between α-laths; however, only the boundaries are highlighted. Due to the HCP structure of α-titanium, polarized light provides a color contrast associated with the crystallographic orientation angle of the individual grain with the surface, highlighting not only grain boundaries but also grains with similar orientation. When using polarized light, etching is not required. The boundaries between α-laths are visible. Parallel laths have similar colors, which indicates a similar crystallographic orientation (confirmed by EBSD). Adjacent β-grains are distinguished by two features: parallel α-laths change direction at prior β-grain boundaries, and α-lath cross-section and color varies. Deep etching of the Ti-6Al-4V layers with nitric acid reveals the morphologies of families of laths. Each lath appears to follow a wedge-shaped pattern (shown in Figure 17) where similar wedge edges are parallel and grow with a leading-edge piercing angle of ~21° (minimum 16° max 30.5°)

### 4.4. The Impact of Successive LENS Thermal Cycles through α + β-Titanium Region

Ahmed and Rack (1998) [40] have shown from Jominy quench experiments on Ti-6Al-4V that aluminum and vanadium segregate during the β- to α-titanium transformation. Although they argued for a rapid, massive diffusionless transformation where solute segregation was limited, they did find by qualitative EDX analysis that aluminum in transformed α-titanium enriched to between 7.6 and 8.3 wt.% and vanadium depleted to between 2.7 and 3.2 wt.% at cooling rates from 20 to 275 °C/s. The significance here is that elemental segregation occurs during a single cooling path through the transformation zone. Banerjee et al. (2003) [43] used the LENS process to deposit a compositionally graded titanium-vanadium alloy where the vanadium composition varied from 0 to 25 wt.%. Using Transmission Electron Microscopy (TEM)-based Energy Dispersive Spectroscopy (EDS) at locations in the deposit with an overall vanadium composition of two atomic percent, Banerjee et al. (2003) [43] observed a segregated depletion of 0.5 atomic percent aluminum in transformed α-titanium laths and 5 atomic percent vanadium enrichment in the inter-lath spaces. The magnitude of vanadium segregation observed by Banerjee et al. (2003) [43] was much greater than that observed by Ahmed and Rack (1998) [40]. However, the LENS process employed by Banerjee et al. (2003) [43] involved multiple thermal cycles passing through the transformation zone, whereas the Jominy quench technique employed by Ahmed and Rack (1998) [40] involved a single cooling transit through the transformation zone.

What is unique about the additive processes? Similar to multi-pass welding processes, both the base material and weld deposit material undergo multiple thermal cycles. For materials that undergo phase transformations, deposited materials can pass through the phase transformation multiple times. For Ti-6Al-4V, the passage of the β- to α-transformation front results in acicular needles when viewed in cross-section, as shown in Figure 15b. As the temperature decreases, we propose that at the β- to α-transformation front, acicular needles transform to the c-axis of the resulting HCP α-plate-like lath. These needles may nucleate along β-to-β interfaces or at existing α/β interfaces. The tapered needle growth consumes the β-grain as adjacent needles grow laterally in the plane of the transformation front. Furthermore, we hypothesize that elemental segregation by diffusion occurs at the interface of the growing α-needle, favoring α stabilizing elements in the needle side of the interface and thus favoring enrichment of β stabilizing elements in the residual β-grain. As α-needles converge, the space in between is enriched by β stabilizing elements. When the temperature increases and the direction of motion of the transformation front reverses, then all previously transformed α-grains revert to the BCC β-structure. While there is a driving force for elemental segregation in the β to α-transformation, there is no segregating driving force from α to β, because aluminum and vanadium are both stable in solid solution with titanium in the BCC crystal lattice above the β-transus temperature. Consequently, any elemental segregation resulting from a β to α-transformation will remain in place unless the temperature increase reaches the homogenization temperature.

## 5. Conclusions

Several conclusions can be made of the chemistry–process–structure relationships of a functionally graded Ti-6Al-4V alloy with boron through a LENS process:

1. A new term, “Borlite”, is proposed related to the liquid–solid transformation eutectic composition of TiB/Ti. The boron affected the solidification characteristics, which in turn determined the primary β-titanium grain size. When the boron wt.% was 0.25%, the primary β-titanium grain size saturated at a minimum level for the process variables in this study.
2. The aluminum and vanadium affected the characteristics of the β- to α-titanium transformation. The transformed titanium α-lath size decreased from 2 μm to 0.2 μm as the aluminum and vanadium content increased from 0 to 6% and 0 to 4%, respectively.
3. The chemistry composition influenced the proper heat depth of penetration to alleviate process pores during deposition. In this case, the chemistry gradients of titanium, aluminum, vanadium, and boron throughout the multilayered LENS build process induced a continuously changing depth of penetration, causing complexities in the control loop of the LENS process and, hence, uncertainties in the multiscale heterogeneous structures.

## Data Availability

The original contributions presented in the study are included in the article, further inquiries can be directed to the corresponding author.
